# Efficacy of levofloxacin, omeprazole, nitazoxanide, and doxycycline (LOAD) regimen compared with standard triple therapy to eradicate *Helicobacter pylori* infection: a prospective randomized study from a tertiary hospital in India 

**Published:** 2021

**Authors:** Hameed Raina, Rajesh Sainani, Arshid Parray, Abdul Haseeb wani, Umaymah Asharaf, Manzoor Ahmad Raina

**Affiliations:** 1 *Department of Gastroenterology and Hepatology, Jaslok Hospital and Research center, Mumbai, India *; 2 *Department of Radiology, SKIMS, Soura, India*; 3 *Government Medical College, Srinagar, India*

**Keywords:** Helicobacter pylori. LOAD regimen, Standard triple therapy, Eradication

## Abstract

**Aim:**

In this study, the efficacy of 14-day triple therapy was compared with that of a novel ten-day LOAD regimen to eradicate *Helicobacter pylori* infection in India.

**Background:**

*Helicobacter pylorus* infection is widespread in India. Resistance to antibiotics commonly used against *Helicobacter pylori* is increasing rapidly, leading to traditional triple therapy's lower success. Therefore, a search for a new regimen is needed

**Methods:**

In this randomized trial, patients with *Helicobacter pylori* infection were randomized to a group receiving LOAD therapy (levoﬂoxacin 250 mg OD, omeprazole 40 mg BD, nitazoxanide 500 mg BD, and doxycycline 100 mg OD) for ten days or a group receiving standard triple therapy (pantoprazole 80 mg, amoxicillin 2000 mg, and clarithromycin 1000 mg daily) in divided doses for 14 days). Gastric biopsy/RUT was done 10–12 weeks after completing therapy to confirm *Helicobacter pylori* eradication.

**Results:**

Eradication rates were significantly greater with the LOAD regimen than with standard triple therapy on both intention-to-treat analysis (82.75% vs. 60.26%, *p* = 0.001; difference, 22.49% [95% CI, 8.5-18%] and per-protocol analysis (83.3% vs. 62.75%, *p* = 0.002; difference, 20.55% [95% CI, 7.1-22.5%]). Both treatment regimens were well tolerated.

**Conclusion:**

Although the rate of eradication of H. pylori infection was significantly higher with the LOAD regimen than triple therapy, the efficacy was still suboptimal, possibly because of fluoroquinolone resistance or the short course of treatment.

## Introduction

*Helicobacter pylori* is a Gram-negative, flagellated, spiral-shaped bacterium that penetrates the mucosal layer of the upper gastrointestinal tract (1). It is associated with chronic gastritis, peptic ulcers, gastric mucosa-associated lymphoid tissue lymphoma, gastric cancer, iron deficiency anemia, etc. (2, 3).* Helicobacter pylori* infection affects about half of the global population. Its prevalence varies among regions due to differences in economic and social conditions in the world (4, 5). In India, the prevalence of *Helicobacter pylori* infection is about 60% (range = 31% to 84%). More than 20 million Indians are believed to have peptic ulcer disease (6). The traditional standard triple therapy (PPI plus clarithromycin and amoxycillin) has been used to eradicate *Helicobacter pylori* for more than 20 years. However, this regimen's treatment success in intention-to-treat (ITT) analysis is below 80%, which in most studies is defined as unacceptable (7).* Helicobacter pylori* resistance rates of this regimen range from 10% to 30% (3). The efficacy is consistently falling below 80%, mainly because of clarithromycin resistance (8). PPI-clarithromycin-containing triple therapy should be abandoned in areas with clarithromycin resistance rates above 15% to 20% (Maastricht IV/Florence Consensus Report, 2012) (9). 

Alternative combination regimens, including bismuth-containing quadruple therapy, concomitant therapy (CT), sequential therapy, hybrid therapy, and quinolone-based triple or quadruple therapies, were explored (10). However, the optimal regimen for *Helicobacter pylori* eradication remains elusive. Therefore, newer treatment regimens aimed at eradicating the organism more eﬀectively are being pursued (11). 

There is an increased demand in India for a more efficacious but tolerable anti-*Helicobacter pylori* treatment because of resistance to metronidazole and clarithromycin in standard therapy. In one of India's multicentric studies conducted by Thyagarajan et al. (12), overall *Helicobacter pylori* resistance rates were 77.9% to metronidazole, 44.7% clarithromycin, and 32.8% to amoxycillin. Multiple resistance was seen in 112/259 isolates (43.2%). Metronidazole resistance was high in Lucknow, Chennai, and Hyderabad (68%, 88.2%, and 100%, respectively) and moderate in Delhi (37.5%) and Chandigarh (38.2%). Ciprofloxacin and tetracycline resistance rates were the least, ranging from 1.0% to 4% (12).

Our hospital is situated in a highly prevalent region of * Helicobacter pylori *infection*. *Many patients with *Helicobacter pylori*-induced gastritis or duodenitis and peptic ulcer disease who require treatment and eradication of the etiological agent are seen in this hospital. However, most of them do not respond to the usual standard triple therapy. Unfortunately, data regarding antibiotic resistance patterns against *Helicobacter pylori* is not available in our institute. Bismuth-containing quadruple therapy has not been widely used in India due to the nonavailability of bismuth. Thus, we were facing many problems in eradicating *Helicobacter pylori* in our patients. To overcome this, we started to prescribe the LOAD regimen to our patients, and we saw excellent results.

We found no study from India that compared the efficacy of the LOAD regimen with that of the standard triple therapy in eradicating *Helicobacter pylori* infection. Therefore, the current study aimed to compare the efficacy of 10 days of LOAD and triple therapy to eradicate *Helicobacter pylori* infection in this part of the world. A novel four-drug regimen, three antibiotics (levofloxacin, nitazoxanide, and doxycycline), and a PPI (omeprazole) to eradicate HP infection in treatment-naive patients were evaluated. 

## Methods

**Study Patients**

Consecutive patients with dyspeptic symptoms undergoing upper endoscopy were recruited for study participation from January 2017 to February 2020 in Jaslok Hospital and Research Hospital, a tertiary care hospital of Mumbai. To be included in the study, patients must have had* Helicobacter pylori*-induced gastritis or duodenitis confirmed on endoscopy with RUT, histopathology, and culture. Patients with active bleeding, age < 20 years, pregnancy, prior HP treatment or infection, current or recent (within six weeks) use of a PPI or H2 receptor antagonists, antacids, anticoagulants, or misoprostol, antibiotics, partial gastrectomy, gastric malignancy, or allergy to any medications used in the study were excluded.

**Diagnosis of** ***Helicobacter pylori***

The detailed clinical profile and endoscopic findings of the patients were recorded. *Helicobacter pylori* infection was diagnosed by serology and rapid urease test (RUT) and confirmed on histology. The RUT (RUT DRY test kit from Gastro Cure System, Kolkata, India) was used as a screening test as it is rapid, cheap, and simple with high sensitivity (88% to 95%), specificity (95% to 100%), positive predictive value (98.75%), negative predictive value (87.5%), and diagnostic accuracy (95%), respectively (13). For RUT, one biopsy was taken from the corpus, one from the incisura angularis, and one from the antrum. Biopsied material was placed in a commercial kit containing urea and a pH indicator. Sharma et al. (13) also used a RUT DRY test kit in their study that showed its sensitivity to be 100% in detecting *Helicobacter pylori* infection (14). The test kit label was peeled back and the biopsied material taken from three sites in the stomach introduced in exposed yellow media, and one drop of distilled water was added. The urease enzyme produced by *Helicobacter pylori* rapidly hydrolyses urea in the kit, producing ammonia. The pH indicator can detect the rise in the pH of the medium by ammonia. RUT was considered positive if media color changed from yellow to red within 3 hours. Multiple biopsies were taken from the antrum, body, and fundus and sent for histopathological examination to confirm gastropathy, duodenopathy, and Pylori infection. Histopathologic evaluation with routine H&E stain has a sensitivity of 70% to 95% that can be increased by special stains such as the silver-based Genta (15). Serology was used in this study to diagnose *Helicobacter pylori* infection, as the study region is a very high prevalence zone, and serology is readily available in the study hospital at a low cost. Furthermore, its use is supported by Dustin et al., who showed that* s*erum *Helicobacter pylori* IgG demonstrated higher sensitivity (0.94) than urea breath and stool antigen tests (0.64 and 0.61, respectively). They concluded that serology's superior sensitivity and negative predictive value support its use as a non-invasive test to rule out *Helicobacter pylori* infection (16).

**Study design**

A randomized, open-label trial was conducted to compare the efficacy and tolerability of a novel four-drug regimen (LOAD) in eradicating *Helicobacter pylori* infection in the Gastroenterology Department of Jaslok Hospital, Mumbai, India. The study compared the LOAD regimen (levoﬂoxacin 250 mg OD, omeprazole 40 mg BD, nitazoxanide 500mg BD, and doxycycline 100 mg OD) for ten days with the current standard of care as represented by the standard triple therapy (STT) containing pantoprazole (40 mg BD); amoxicillin (1 gm BD), and clarithromycin (100 mg BD) for two weeks. Before randomization, patients underwent a washout period of 6 weeks from any antibiotic or PPI use, and written informed consent was obtained from them before the initiation of therapy. Patients with *Helicobacter pylori* infection were randomized into two groups using a computer-generated random number table. One group received a LOAD regimen comprising levoﬂoxacin 250 mg OD, omeprazole 40 mg BD, nitazoxanide 500 mg BD, and doxycycline 100 mg OD for ten days. Another group received standard triple therapy comprising pantoprazole 80 mg, amoxicillin 2000 mg, and clarithromycin 1000 mg daily in divided doses. PPI was continued for eight weeks post-completion of anti*-Helicobacter pylori* treatment in all patients. Patients were followed in OPD every week. Compliance and adverse effects were assessed weekly throughout the study in all patients.

A pill intake of more than 90% was considered as good compliance. A follow-up endoscopy was done 10–12 weeks after completing *Helicobacter pylori* treatment, and a RUT test and gastric biopsies were taken for histology to confirm *Helicobacter pylori* eradication. PPI was stopped at least two weeks before the follow-up endoscopy. A stool antigen test was not done, as the facility was not available in the study institute. Furthermore, customs and habits in India were obstacles to delivering fresh stool on time to outside diagnostic centers.

**Figure 1:**
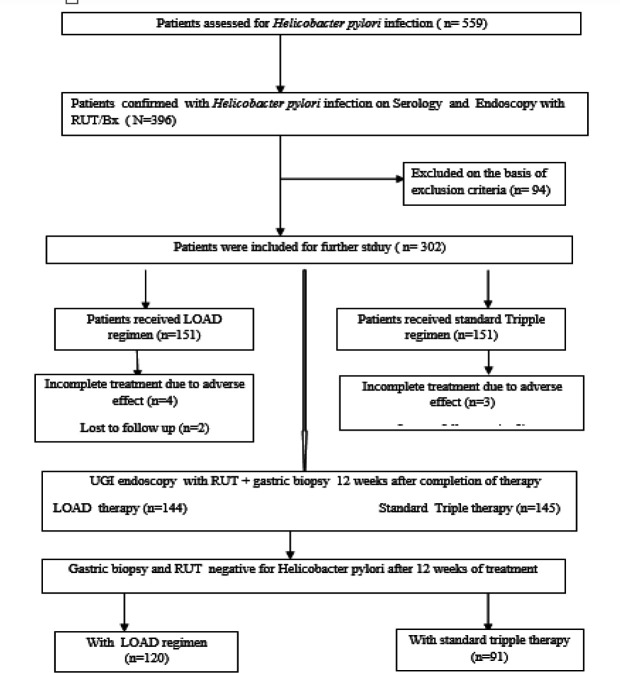
Flow chart of the study

Additionally, qualitative stool antigen test kits have low sensitivity and use polyclonal antibodies, making them less favorable (17). Patients readily consented to the check endoscopy being readily available, cost-effective, and more productive than other tests. Check endoscopy also helped evaluate the *Helicobacter pylori* treatment response concerning ulcer healing, gastropathy, duodenopathy, and esophagitis (Figure 1).

**Outcome assessments**

**Primary outcome**

The primary outcome was the eradication of *Helicobacter pylori* infection. Eradication was defined as negative RUT and negative histology or culture for *Helicobacter pylori.*

**Secondary outcome**

Secondary outcome measures were compliance and adverse effects to the therapy. 

**Statistical analysis**

Comparisons between the two groups were made using the independent sample t-test for quantitative variables. Eradication rates were calculated both by “intention-to-treat” (ITT) and “per protocol” (PP) analyses. In ITT analysis, all enrolled patients were included. In PP analysis, only compliant patients who underwent follow-up gastric biopsy after completing therapy to confirm *Helicobacter pylori* eradication were considered. A *p*-value < 0.05 was considered statistically significant. 

**Ethical approval**

According to the Declaration of Helsinki, the Institutional Ethics Committee of Jaslok Hospital and Research Institute, Mumbai, approved the study (OR/JHR/IEC- 674- 2017). Signed informed consent was obtained from each patient before enrollment in the study, and separate consent was obtained for endoscopy. 

## Results

**Demographics**

Five hundred fifty-nine patients who presented with GI symptoms between February 2015 and January 2020 were initially evaluated for the study. Men comprised 53.66% and women 46.33% of the patients. Mean patient age was 38.8 ± 10.2 years, and age range was from 14 to 75 years. Reported symptoms were dyspepsia in 353 (63.14%), early satiety in 68 (12.16%), gastroesophageal reﬂux with dyspeptic symptoms in 59 (10.55%), nausea/vomiting in 41 (7.33%), loss of appetite in 23 (4.11%), and abdominal pain in 15 (2.68%). All participants underwent esophagoduodenoscopy (EGD) in the endoscopy suite with a GIF Q170 UGI Endoscope. Of these patients, 396 (70.84%) had confirmed HP gastropathy, duodenopathy, or both on endoscopy and histopathology or culture. Ninety-four patients (23.73%) were excluded based on the exclusion criteria. The main findings on endoscopy were gastritis (46.35%), predominantly antral followed by multifocal. Isolated duodenal involvement, predominantly duodenitis, was present in 23.84% of patients. Both gastric and duodenal involvement was seen in 20.52% of patients and esophagitis in 7.28% of patients (Table 1).

**Primary outcomes**

The study results showed eradication rates of 83.33% (120/144) with the LOAD regimen and only 62.75% (91/145) with the standard triple therapy regimen; the difference is 20.55% [95 % CI, 7.1-22.5 %] (*p*=0.002). Intention-to-treat (ITT) analysis also revealed that eradication rates were 82.75% (120/151) with the LOAD regimen and 60.26% (91/151) with standard triple therapy (STT) regimen, the difference being 22.49% [95% CI, 8.5-18%] (*p*=0.001) (Table 1).

**Secondary outcomes**

No diﬀerence in patient adherence among LOAD and standard regimens was detected. Overall, 95.36% (144/151) and 96.02% (145/151) of patients completed the LOAD and standard triple therapy regimens, respectively, which is considered very good. In the LOAD group, four patients discontinued therapy owing to severe gastrointestinal distress (n=2), dizziness (n=1), or palpitations (n=1), and two patients were lost to follow up. Three patients in the standard arm discontinued therapy secondary to diarrhea (n=2) and gastrointestinal distress (n=1), and two patients were lost to follow up (Table 2).

No diﬀerence was observed in reported adverse events between the groups, except diarrhea which was significantly more with the standard group (*p*=0.023) (Table 3).

## Discussion

In response to rising resistance rates, therapies including concomitant quadruple therapy and sequential therapy have emerged as alternatives to standard therapy (18-20). Although sequential therapy is innovative, the regimen is also very complex, requiring mid-therapy transition from dual to triple therapy (21).

The current study indicates that a unique four-drug regimen (LOAD) is potentially more efficacious than the standard of care for the treatment of HP in a treatment-naive population. Overall, patients receiving standard triple therapy had an unsatisfactory cure rate of 73.3% ITT (77.6% per protocol); this outcome is similar to those reported by other studies (7). 

**Table1:** Baseline parameters of the patients

Parameters	Values
Mean Age in years ( range 14 to 75 years )	38.8 ± 10.2 years
Sex (male to female) (numbers)	300 (53.66%) to 259 (46.33%)
Dyspepsia	353 (63.14%)
Early satiety	68 (12.16 %)
Gastroesophageal reflux withdyspepsi	59(10.55 %
Nausea/Vomiting	41( 7.33%)
Loss of appetite	23(4.11 %),
Abdominal pain	15 (2.68 % )
Confirmed H.pylori Gastropathy/duodenopathy on RUT /Bx	396(70.84%)

**Table 2:** Endoscopic features among the two groups

Endoscopic features	LOAD regimen group ( n= 151)	Standard triple therapy (n=151)	P value
Isolated gastric lesion (%) Gastric ulcer Antral gastritis Corporal gastritis Fundal gastritis Multifocal gastritis	70 (46.35) 11 23 12 4 20	72 (47.68) 12 25 10 5 18	0.62
Isolated duodenal lesion (%) Duodenal ulcer Duodenitis	36 (23.84) 8 28	32 (21.19) 10 26	0.31
Combined duodenal and gastric lesion (%)	31 (20.52)	33 (21.85)	0.67
Esophagitis (%)	11 (7.28)	12 (7.94)	0.81
Normal UGI endoscopy	3 (1.98)	2 (1.32)	0.72

**Table 3:** Eradication Rates of Helicobacter pylori infection between the groups

	Eradication achieved/analysed	Eradication rate %	P value
Per protocol therapy			0.001
Standard tripple therapy	91 / 145	62.75	
Modified LOAD therapy	120/144	83.33%	
Intention to treat analysis (ITT)			0.002

Standard tripple therapy	91/151	60.26	
Modified LOAD therapy	120/151	82.75	

On the other hand, the LOAD cure rate of 89.4% ITT (93.6% per protocol) meets the criteria that content experts deem acceptable (22, 23). The antibiotics used in this study (levoﬂoxacin, doxycycline, and nitazoxanide) were selected because of ease of dosing (once or twice daily) and supportive data indicating these agents are highly active against HP (24). A pilot study in patients previously failing treatment successfully used a similar regimen, LEND (levoﬂoxacin, esomeprazole, nitazoxanide, and doxycycline), to achieve a comparable ITT eradication rate of 90% (27/30) (25). Levoﬂoxacin, a bactericidal ﬂuoroquinolone antibiotic, has activity against HP due primarily to the drug’s activity on bacterial DNA gyrase (26). Levoﬂoxacin has been advocated for use in second- and third-line “rescue” regimens (27, 28). Unfortunately, ﬂuoroquinolone resistance, especially in patients who have routinely received a ﬂuoroquinolone for other indications, is of particular concern (29, 30). 

Doxycycline, a tetracycline analog, has bacteriostatic properties and inhibits bacterial protein synthesis (31). The current literature indicates little to no resistance to this class (32, 33), thus supporting the use of tetracyclines as superior alternatives for rescue therapies (34). 

Although tetracycline is traditionally dosed four times a day, doxycycline oﬀers the advantages of once- or twice-daily dosing, thus potentially improving compliance and tolerability (35). In the current study, LOAD therapy took advantage of doxycycline's prolonged half-life by using a single 100 mg dose at dinner. Dosing doxycycline only once a day may increase tolerability while decreasing pill burden, but, as in the case with levoﬂoxacin, it is possible that twice-daily dosing may improve clinical outcomes. Although the current study showed the higher efficacy of the LOAD regimen (83.33%) as compared to standard therapy (62.75%), the response rate was still considered suboptimal (24, 25). Basu et al. (8) found higher eradication rates with LOAD regimens. Their intention-to-treat analysis revealed significant differences (*p*<0.05) in the respective eradication rates of the LOAD therapies, 88.9% (80/90) with LOAD-10, 90% (81/90) with LOAD-7, and 89.4% (161/180) for combined LOAD compared with those receiving LAC, 73.3% (66/90). One reason for this may be higher resistance to levofloxacin in the population of the current study.

No published study from India was found that compared the efficacy of the LOAD regimen with triple therapy to eradicate *Helicobacter pylori* infection. Two studies from India were found which compared the efficacy of bismuth-containing quadruple therapy with triple therapy for eradication of *Helicobacter pylori* infection. These studies showed the similar efficacy of both regimens (36, 37). Recently, one study compared the efficacy of concomitant therapy with sequential therapy. Both regimens showed similar efficacy to eradicate *Helicobacter pylori* infection (38).

Therefore, the current study is the first to show an alternative regimen more efficacious and well-tolerated than the standard of care for *Helicobacter pylori* treatment, at least in Indian patients. However, this study has certain limitations: 1. Data regarding *Helicobacter pylori's* antibiotic resistance pattern is not available in our institute and could not be utilized in this study. 2. Invasive tests (endoscopy) were used to confirm *Helicobacter pylori* eradication rather than non-invasive tests, which may not be feasible or approved in other places. 3. The long-term follow-up/recurrence of these patients was not studied.

Hence, considering the suboptimal response of LOAD therapy in the current study, better information on resistance patterns and eradication rates in local populations is critical for choosing effective regimens.

The eradication rate of *Helicobacter pylori* was significantly higher with the LOAD regimen than the triple therapy group. However, the response rate is still suboptimal. Perhaps higher doses or a longer duration would be able to overcome HP infection. To determine that, further studies are needed.

## Conflict of interests

The authors declare that they have no conflict of interest.
